# Amelioration of Alcohol Induced Gastric Ulcers Through the Administration of *Lactobacillus plantarum* APSulloc 331261 Isolated From Green Tea

**DOI:** 10.3389/fmicb.2020.00420

**Published:** 2020-03-17

**Authors:** Haryung Park, Donghyun Cho, Eunchong Huang, Ju Yeon Seo, Wan Gi Kim, Svetoslav Dimitrov Todorov, Yosep Ji, Wilhelm Heinrich Holzapfel

**Affiliations:** ^1^Advanced Green Energy and Environment Institute (AGEE), Handong Global University, Pohang, South Korea; ^2^Vital Beautie Research Division, Amore Pacific R&D Unit, Gyeonggi-do, South Korea

**Keywords:** alcohol induced gastric ulcers, *Lactobacillus plantarum*, short chain fatty acids, gut microbiome, *Akkermansia*

## Abstract

Gastric inflammation is an indication of gastric ulcers and possible other underlying gastric malignancies. Epidemiological studies have revealed that several Asian countries, including South Korea, suffer from a high incidence of gastric diseases derived from high levels of stress, alcoholic consumption, pyloric infection and usage of non-steroidal anti-inflammatory drugs (NSAIDs). Clinical treatments of gastric ulcers are generally limited to proton pump inhibitors that neutralize the stomach acid, and the application of antibiotics for *Helicobacter pylori* eradication, both of which are known to have a negative effect on the gut microbiota. The potential of probiotics for alleviating gastrointestinal diseases such as intestinal bowel syndrome and intestinal bowel disease receives increasing scientific interest. Probiotics may support the amelioration of disease-related symptoms through modulation of the gut microbiota without causing dysbiosis. In this study the potential of *Lactobacillus plantarum* APSulloc 331261 (GTB1^TM^), isolated from green tea, was investigated for alleviating gastric inflammation in an alcohol induced gastric ulcer murine model (positive control). Treatment with the test strain significantly influenced the expression of pro-inflammatory and anti-inflammatory biomarkers, interleukin 6 (IL6) and interleukin 10 (IL10), of which the former was down- and the latter up-regulated when the alcohol induced mice were treated with the test strain. This positive effect was also indicated by less severe gastric morphological changes and the histological score of the gastric tissues. A significant increase in the abundance of *Akkermansia* within the GTB1^TM^ treated group compared to the positive control group also correlated with a decrease in the ratio of acetate over propionate. The increased levels of propionate in the GTB1^TM^ group appear to result from the impact of the test strain on the microbial population and the resulting metabolic activities. Moreover, there was a significant increase in beta-diversity in the group that received GTB1^TM^ over that of the alcohol induced control group.

## Introduction

Gastric ulcers, also known as peptic ulcers, develop within the epithelial lining of the stomach due to the reduction of the mucus layer by the enhanced secretion of acid and pepsin. Some of the main contributing factors to these erosion processes within the stomach are *Helicobacter pylori* infection, abuse of alcohol, smoking, or the prolonged use of non-steroidal anti-inflammatory drugs (NSAIDs) (Toljamo et al., 2011). According to the systematic review on the global incidence of peptic ulcers by Azhari et al. (2018), South Korea has one of the highest annual incidences of perforated peptic ulcers in the 21^st^ century. The clinical approaches for treatment are still limited to proton pump inhibitors or the eradication of *H. pylori*, that includes antibiotic therapy. Most of the recommended drugs, such as the proton pump inhibitors prevent gastric ulcers through the suppression of acid production within the stomach, disrupting the protective pH barrier which promotes a higher risk of enteric infections caused by bacterial pathogens such as *Clostridium difficile*, *Salmonella* spp. and *Campylobacter* spp. (Imhann et al., 2017). Therefore, other alternative remedies are needed to counteract gastric ulcers by also protecting the gut from enteric infections.

Different strains of the lactic acid bacteria (LAB), and particularly some *Lactobacillus* spp., are representing some of the most commonly used probiotics, defined as “live microorganisms which, when administered in adequate amounts, confer a health benefit on the host” (Food and Agriculture Organization [FAO], and World Health Organization [WHO], 2002). Extensive research has been performed on the efficacy of various *Lactobacillus* strains on gastric ulcers (Dharmani et al., 2013; Khoder et al., 2016) either as single therapeutic agents or in combination with antibiotics (Boltin, 2016; Goderska et al., 2018). The majority of the investigated strains have been shown to inhibit or protect the gastric mucosal barrier through the up-regulation of prostaglandin E2 (Uchida and Kurakazu, 2004; Gotteland et al., 2006; Lam et al., 2007; Uchida et al., 2010), enhancement of mucus secretion (Gomi et al., 2013) or the regulation of inflammatory responses (Konturek et al., 2009; Şenol et al., 2014). Moreover, not only do these probiotics have prophylactic effects, but some of them also exert therapeutic effects through the enhancement of epithelial growth (Singh and Kaur, 2012), promotion of angiogenesis (Dharmani et al., 2013) and up-regulation effect on anti-inflammatory cytokines expression (Virchenko et al., 2015). Currently, approaches involving therapies either with “new” antimicrobials and/or probiotics are considered promising approaches for therapeutics or prophylactics of gastric ulcers (Goderska et al., 2018).

Several studies suggest that possible underlying mechanisms for the effect of probiotics on the host physiology may be related to their modulating role on the gut microbiota (Delzenne et al., 2011; Gerritsen et al., 2011; Gomes et al., 2014; Park et al., 2017; Ji et al., 2018). Yet, a deeper understanding of the impact of probiotic administration on ameliorating gastric ulcers is still lacking and needs further investigation and better understanding. Current knowledge is suggesting that butyrate, one of the three major short chain fatty acids (SCFAs) produced by the intestinal microbiota, has a protective effect against ethanol-induced gastric ulcer formation. Pre-treatment with butyrate down-regulated the pro-inflammatory cytokines IL1β, TNFα, and IL6 and enhanced the gastric wall mucus (Liu et al., 2016). Furthermore, a butyrate-producing *Clostridium butyricum* showed a similar effect as sodium butyrate in the alcohol induced gastric ulcer model and also alleviated the gastric mucosal damage and inflammation within the cold stress model and the pyloric induction model (Wang et al., 2015). A recent publication also demonstrated the protective effect of acetate against ethanol-induced acute gastric ulcers through the regulation of inflammation in the gastric mucosa (Liu et al., 2017). Even when the efficacy of probiotics, butyrate-producing species and short chain fatty acids on gastric erosion has been reported (Wang et al., 2015; Liu et al., 2017), data obtained in studies on the relationship between these administrations and the modulation of the gut microbiota in alcohol erosion models need further critical assessment in support of a better understanding on protective processes.

Probiotics are known to modulate the gut microbiota which may in turn regulate the production of the SCFAs within the gut. Therefore, in this study we explored the efficacy of *Lb. plantarum* APSulloc 331261 GTB1^TM^ by focusing on the production of SCFAs derived from the modulation of the gut microbiota in alcohol induced gastric ulcers.

## Materials and Methods

### Bacterial Culture Condition

*Lactobacillus plantarum* APSulloc 331261, isolated from green tea (Dolsongi tea field, Jeju island, South Korea) was provided by Amore Pacific (KCCM11179P), and its safety and beneficial properties were described previously (Arellano et al., 2019). Recently, the designation of this strain has been amended with its trademark (GTB1^TM^), and further reference to this strain (*Lactobacillus plantarum* APSulloc 331261 GTB1^TM^), will be as GTB1^TM^. *Lb. plantarum* 299v is a commercial probiotic strain that has been investigated widely for its beneficial physiological functions, amongst others for improving irritable bowel syndrome (Ducrotté et al., 2012). The *Lb. plantarum* strains (GTB1^TM^ and 299v) were cultured at 37°C for 18 h and prepared on a daily basis in MRS broth (Difco Laboratories Inc., Franklin Lakes, NJ, United States). Bacterial cell suspensions for experimental purposes were obtained by centrifugation of overnight cultures (12,000 × *g* for 1 min at 4°C), the pellet was washed twice with sterile PBS (1x, pH 7.4, Lonza^TM^BioWhittaker^TM^, Walkersville, MD, United States) and re-suspended in PBS to match the concentration of 1 × 10^9^CFU per 200 μL of PBS before administration to the mice.

*Helicobacter pylori* SS1 (HpKTCC), used to induce a pyloric infection in murine models, was grown on Brucella broth (MBcell, Seoul, South Korea), supplemented with 1.5% agar mixed with 10% bovine serum at 37°C in presence of 5% CO_2_ with high humidity and sub-cultured every 2∼3 days (Panasonic Incubator, Osaka, Japan).

### Murine Models

All procedures carried out in the animal study were approved by the Animal Ethical Committee of Handong Global University, South Korea (20160616-007). Four-week-old, specific pathogen free (SPF), Institute for Cancer Research (ICR) male mice obtained from Saeronbio Inc. (Gyeonggi-do, South Korea) were provided with sterilized water and normal chow diet (Purina, Chicago, IL, United States) at *ad libitum* and housed under 23°C ± 1°C and 55 ± 10% humidity in a 12 h light/dark cycle.

### Alcohol Induced GU Murine Model

All the mice were adapted in the set environment for 1 week and then randomized into five groups (*n* = 8) as described: Group A – PBS (control group without ethanol induction); Group B – EtOH (control group with ethanol induction); Group C – AP (*Lb. plantarum* APSulloc 331261 GTB1^TM^); Group D – 299v (probiotic control, *Lb. plantarum* 299v); Group E – OMPZ (most commonly used proton pump inhibitor drug control, Omeprazole).

The probiotics groups, receiving GTB1^TM^ and *Lb. plantarum* 299v, respectively, were pretreated with 1 × 10^9^ CFU/day, for 7 days before ethanol induction, while the omeprazole group was fed with 13 mg/kg body weight/day of omeprazole according to the dose applied by Wang et al. (2015), for the same period. After the pre-treatments, the alcohol induced GU model was prepared and performed according to Liu et al. (2016) on 8 days and the mice were sacrificed 1 h after the induction. Part of the gastric tissue was fixated in 4% formaldehyde for histological assessment and the serum, cecum, liver, and stomach were collected and stored at −80°C until further analysis.

### Ethanol Erosion and *H. pylori* Infection Murine Model

Following the initial induction with ethanol and *H. pylori* infection, the groups received the probiotic strains (*Lb. plantarum* GTB1^TM^ and 299v) and the drug Omeprazole as control; sacrifice followed 2 weeks later. The 60% ethanol was fed orally to the pre-treatment and post-treatment groups 3 h prior to *H. pylori* infection by re-suspending 2 × 10^9^ CFU/mL PBS and administering 0.5 mL to each animal according to Lee et al. (2006). All mice were sacrificed 2 weeks after the ethanol induction and *H. pylori* infection. Part of the gastric tissue was fixed in 4% formaldehyde for histological assessment and the serum, cecum, liver, and stomach were collected and stored at −80°C until further analysis.

### H&E Staining of Gastric Tissue

The paraffin blocks of the gastric tissues fixed in 4% formaldehyde were analyzed by the Korea Experimental Pathology Inc. (Gwangju-si, Gyeonggi-do, South Korea). These tissues were sectioned into 7 μm thick specimens and stained with hematoxylin and eosin (H&E). Observations were made under a light microscope to determine levels of pathological damage. The tissues were blindly scored according to gastric mucosal injury and neutrophil infiltration at a scale of 0–4 provided by Erben et al. (2014).

### RNA Extraction and qRT-PCR Analysis

Gastric tissue was homogenized in 1 mL of TRIzol^TM^ Reagent (Invitrogen^TM^, Thermo Fisher Scientific, San Diego, CA, United States) and RNA extracted according to the manufacture’s protocol. SPECTROstar Nano (BMG Labtech, Offenburg, Germany) was used to evaluate the RNA concentration and purity. Two micrograms of complementary DNA were prepared using the GoScript^TM^ Reverse Transcription System (Promega, Madison, WI, United States) and 5 min incubation with oligo-dT primer at 70°C on the Verity 96-well thermal cycler (Applied Biosystems, Foster City, CA, United States). Specific primers for analyzing gastric cytokines (Supplementary Table S1), SYBR green Premix Ex Taq^TM^ II (Takara, Shiga, Japan) and 20 ng of cDNA were mixed for each reaction using the Step-One Plus real-time PCR system (Applied Biosystems) for quantitative real-time PCR analysis according to Park et al. (2017).

### Cecal Short Chain Fatty Acid (SCFA) Detection

The SCFAs, obtained from 40 mg of cecal content, were mixed with 150 μL of extraction buffer formulated according to Schwiertz et al. (2010) and lysed for 3 min in a Mini-Beadbeater-16 (BioSpec Products, Bartlesville, OK, United States). The mixture was incubated in a horizontal shaker at 25°C for 1 h and then centrifuged at 16,000 × *g* and 25°C for 5 min. The supernatant was carefully transferred into a 9 mm blue screw-capped clear glass vial with interlock insert (Agilent Technologies, Palo Alto, CA, United States). Volatile Free Acid Mix (Supelco, 46975-U, Sigma-Aldrich, St. Louis, MO, United States) was used to create a standard curve for the quantification of the cecal SCFAs that were detected using gas chromatography (Shimadzu GC2010, Shimadzu, Kyoto, Japan).

### Cecum DNA Extraction and qRT-PCR Analysis

DNA was extracted by using the QIAmp DNA mini-kit (Qiagen, Valencia, CA, United States). 50 mg of the cecum were mixed with 0.3 g of 0.1 mm sterile zirconia beads with 700 μL of ASL Buffer (Qiagen) in a 2 mL bead beating tube and homogenized for 3 min in the Mini-Beadbeater-16. The following DNA extraction procedures were performed according to the QIAmp DNA Mini Kit manufacturer’s instructions. Microbiota analysis of the supplementary model was performed by qRT-PCR and using the specific primers suggested by Bergström et al. (2012).

### Cecum Library Preparation and Microbiota Analysis

Cecal genomic DNA was diluted with 10 mM Tris–HCl pH 8.5 buffer to 5 ng/μL prepared according to the Illumina 16S metagenomics sequencing library protocol. The 16S rRNA V3-V4 region was amplified using the following amplicon primers: 16S Amplicon PCR Forward Primer 5′TCG TCG GCA GCG TCA GAT GTG TAT AAG AGA CAG CCT ACG GGN GGC WGC AG′3 and 16S Amplicon PCR Reverse Primer 5’GTC TCG TGG GCT CGG AGA TGT GTA TAA GAG ACA GGA CTA CHV GGG TAT CTA ATC C′3.

The amplified samples with linker primers were then barcoded using the dual indexing method involving the Nextera XT kit (Illumina, San Diego, CA, United States). The final products were normalized and pooled using PicoGreen, and the size of libraries were verified using the LabChip GX HT DNA High Sensitivity Kit (PerkinElmer, Waltham, MA, United States) and performed on an Illumina Miseq platform.

The barcode, linker, and primer sequences were then removed from the original sequencing reads and were replaced with sample names. The removed reads were then merged by their paired-ends using FLASH v 1.2.11. The merged reads containing two or more ambiguous nucleotides, those with a low-quality score (average score < 20), and reads shorter than 300 base pairs, were filtered out. Potential chimeric sequences were detected using the Bellerophon method. The pre-processed reads from each sample were used to calculate the number of operational taxonomic units (OTUs). The number of OTUs was determined by clustering the sequences from each sample using a 97% sequence identity cut-off and the taxonomic profiling and diversity of each group were analyzed and visualized using macQIIME software (v.1.9.1).

### Statistics

All values are expressed as mean ± SD. Statistical differences were determined by one-way ANOVA using Fisher’s Least Significant Difference (LSD). A *p*-value less than 0.05 was considered statistically significant.

## Results

### Efficacy of *Lb. plantarum* APSulloc 331261 GTB1^TM^ Against Alcohol Induced Gastric Ulcer

**FIGURE 1:**
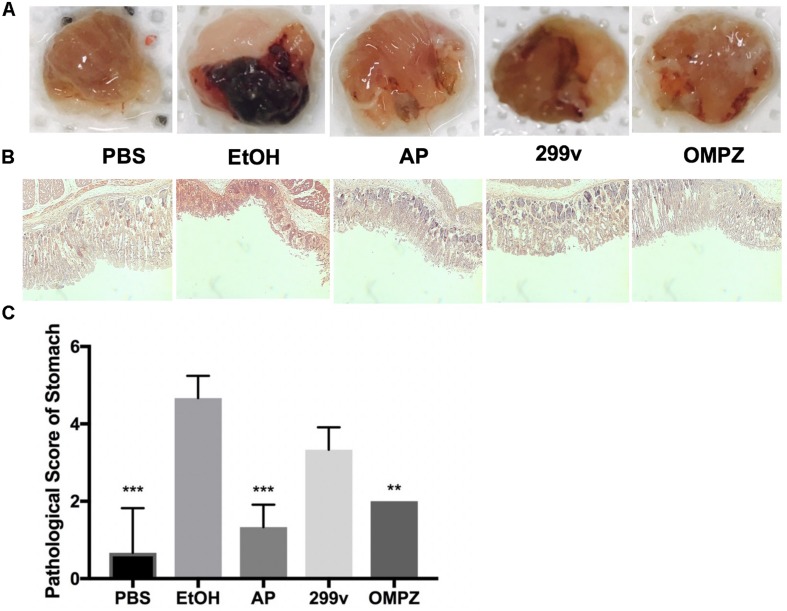
Effects of *Lactobacillus plantarum* APSulloc 331261 GTB1^TM^ on the macroscopic morphology, and histological assessment of acute gastric injuries of the gastric mucosa of ethanol challenged mice. **(A)** Gross morphology of the gastric mucosa of mice challenged with ethanol, **(B)** H+E staining of the gastric mucosa, **(C)** pathological analysis of the gastric mucosal histology. PBS: non-ethanol treated group, EtOH: ethanol treated control group, OMPZ: omeprazole treated group, 299v: *Lactobacillus plantarum* 299v, AP: *Lactobacillus plantarum* APSulloc 331261 GTB1^TM^. **p < 0.01, ***p < 0.005.

The visual damage shown from the gross morphology of the gastric mucosa was significantly different in the GTB1^TM^ treated group (AP) after the induction with alcohol, and showed lower impairment of hemorrhaging gastric tissue (Figure 1A). Furthermore, histological staining revealed less mucosal tissue damage while the pathological score in the AP fed group was significantly lower than that of the ethanol induced (EtOH) group (Figures 1B,C).

### Expression of Inflammatory Cytokines in Alcohol Induced Gastric Tissue

**FIGURE 2:**
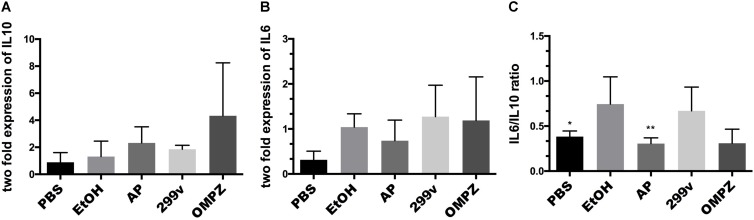
The effect of *Lactobacillus plantarum* APSulloc 331261 GTB1^TM^ on IL10 and IL6 levels in gastric tissue of the ethanol induced GU mice model **(A)** expression of interleukin 10 (IL10) **(B)** expression of interleukin 6 (IL6) **(C)** IL6/IL10 ratio. PBS: non-ethanol treated group, EtOH: ethanol treated control group, OMPZ: omeprazole treated group, 299v: *Lactobacillus plantarum* 299v, AP: *Lactobacillus plantarum* APSulloc 331261 GTB1^TM^. Data was analyzed with one-way-ANOVA compared to EtOH *p < 0.05, **p < 0.01.

There was no significant difference in the AP (GTB1^TM^) group compared to the EtOH group in the expression levels, neither of the anti-inflammatory cytokine interleukin 10 (IL10) nor the pro-inflammatory cytokine interleukin (IL6) (Figures 2A,B). However, the ratio of IL6/IL10 showed a significant difference with the EtOH group in both the non-treated PBS control and AP (GTB1^TM^) groups (Figure 2C). Furthermore, in the study of the *H. pylori* infected mice after erosion of the gastric mucosa through alcohol induction, the inflammatory cytokines tumor necrosis factor alpha (TNF-α), interleukin 1 beta (IL1β) and interleukin 4 (IL4) were significantly down-regulated in the GTB1^TM^ treated mice when compared to the HP control (*H. pylori* infected) group and, with the exception of TNF-α, also the 299v group (Supplementary Figure S1).

### Effects of *Lb. plantarum* APSulloc 331261 on SCFA Production

**FIGURE 3:**
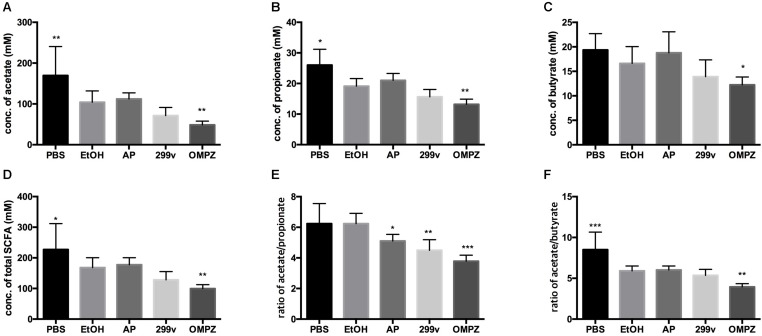
Short chain fatty acids (SCFA) in the ethanol treated mouse cecum: **(A)** acetate **(B)** propionate **(C)** butyrate **(D)** total SCFA **(E)** ratio of acetate/propionate **(F)** ratio of acetate/butyrate. PBS: non-ethanol treated group, EtOH: ethanol treated control group, OMPZ: omeprazole treated group, 299v: *Lactobacillus plantarum* 299v, AP: *Lactobacillus plantarum* APSulloc 331261 GTB1^TM^. Data was analyzed with student’s *t*-test and compared to EtOH treatment; *p < 0.05, **p < 0.01.

While no significant difference in the production of the SCFAs acetate, propionate and butyrate could be detected between the positive control (EtOH treated group) and the two LAB treated groups, OMPZ treatment resulted in a significant decrease in each of the three SCFAs (Figures 3A–C) and the total SCFAs (Figure 3D). On the other hand, a significant decrease was recorded in the acetate/propionate ratio in the LAB treated and the omeprazole drug control (OMPZ) groups (Figure 3E) and also in the acetate/butyrate ratio (Figure 3F). Moreover, in the study on the *H. pylori* infected mice, all three SCFAs significantly increased only in the post-treatment GTB1^TM^ and OMPZ groups after erosion of the gastric mucosa through alcohol induction (Supplementary Figure S2).

### Modulation of the Gut Microbiota Through the Administration of *Lb. plantarum* APSulloc 331261 GTB1^TM^

**FIGURE 4:**
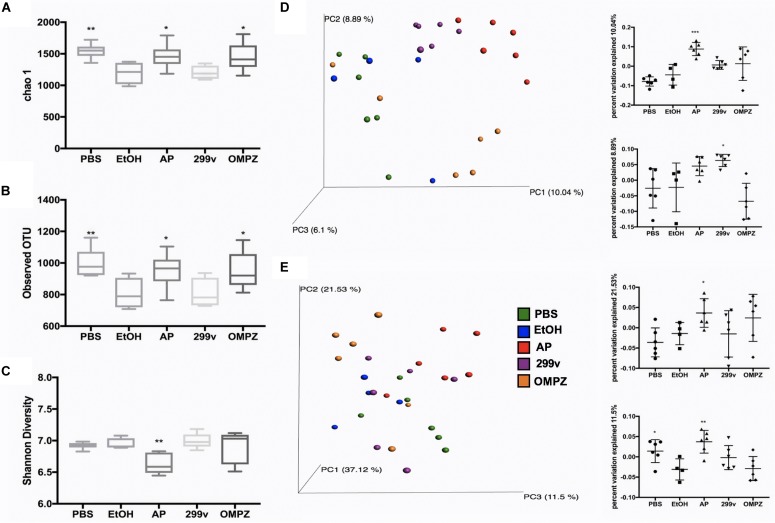
Alpha and beta diversity of cecal microbiota from ethanol treated mice: **(A)** Chao 1, **(B)** Observed OTUs, **(C)** Shannon Diversity, **(D)** unweighted beta-diversity principal coordinates analysis and value of PC1 and PC2 principle coordinate dimensions, and **(E)** weighted beta- diversity principal coordinates analysis and value of PC2 and PC3 principle coordinate dimensions. PBS: non-ethanol treated group, EtOH: ethanol treated control group, OMPZ: omeprazole treated group, 299v: *Lactobacillus plantarum* 299v, AP: *Lactobacillus plantarum* APSulloc GTB1^TM^. Data was analyzed with one-way-ANOVA compared to EtOH *p < 0.05, **p < 0.01.

**FIGURE 5:**
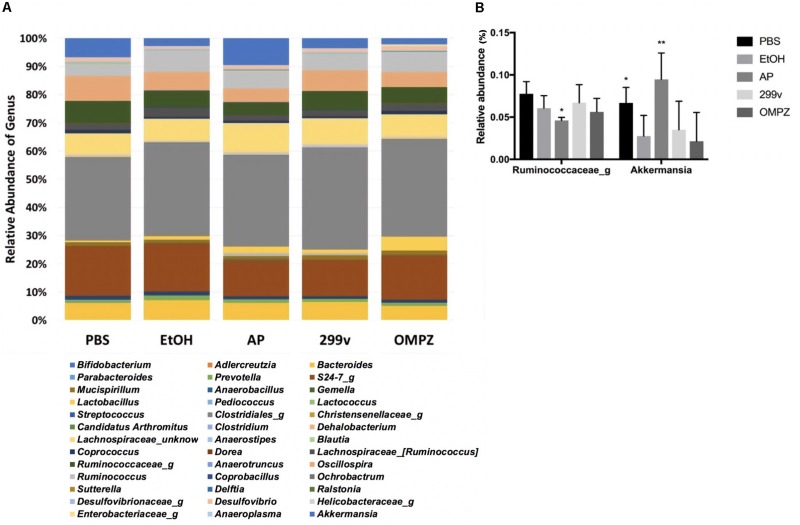
Taxonomic analysis of cecal microbiota from ethanol treated mice: **(A)** relative abundance of the genus level taxonomic composition, and **(B)** relative abundance of the genera *Akkermansia* and *Ruminococcus.* PBS: non-ethanol treated group, EtOH: ethanol treated control group, OMPZ: omemprazole treated group, 299v: *Lactobacillus plantarum* 299v, AP: *Lactobacillus plantarum* APSulloc GTB1^TM^. Data was analyzed with one-way-ANOVA compared to EtOH *p < 0.05, **p < 0.01.

The administration of GTB1^TM^ induced significant changes in the structure of the gut microbiota and the ratio among bacterial groups. Both the un-weighted and weighted beta diversity showed a significant shift in the microbial community when compared to the alcohol-induced (EtOH) control (Figures 4D,E). A clear distinction between the microbial communities is shown across the PC1 of the un-weighted principle coordinate analysis (PCoA) plot (Figure 4D) and PC1 and PC2 of the weighted PCoA plot (Figure 4E). Furthermore, the AP group showed significantly higher richness in alpha diversity (Chao 1 and observed OTUs) but lower evenness (Shannon diversity) compared to the EtOH control group (Figures 4A–C). At the genus level there was a significant increase of *Akkermansia* in the AP group compared to the EtOH group, while in the family *Ruminococcaceae* one genus was significantly decreased in the AP group (Figure 5). It is also noteworthy that, compared to all other groups, administration of GTB1^TM^ resulted in a significant increase in abundance of both *Bifidobacterium* spp. and *Clostridium butyricum*. More detailed information on modulation of the gut microbiota resulting from the different treatments is shown in Supplementary Figure S3.

## Discussion

Overconsumption of alcohol is known to impair the gastric mucosal barrier causing extensive hemorrhagic injuries, accumulate oxidative stress and increase inflammation through the production of cytokines such as IL1β, IL6, and TNF-α (Liu et al., 2017). The excessive use of alcohol not only causes organ dysfunction and health problems, but also disrupts the stability of the intestinal microbiome, resulting in what is generally known as dysbiosis (Engen et al., 2015). Some cases of microbial dysbiosis were associated with a decrease of *Verrucomicrobiae* and *Bacteriodes* and an increase in *Gammaproteobacteria* and *Bacilli* in alcoholics (Chen et al., 2011; Mutlu et al., 2012). In alcohol induced dysbiotic mice the *Proteobacteria* and *Actinobacteria* populations increased and were contrasted by a decrease in *Bacteroidetes*. On the other hand, the treatment of probiotics induced a shift in the three phyla in the opposite direction (Bull-Otterson et al., 2013). Furthermore, beneficial effects through the direct consumption of SCFAs, known as microbial by-products, have previously been proved effective in alcohol induced murine models (Liu et al., 2016, 2017). In pilot studies several attempts have been made for the direct delivery of SCFAs as postbiotic, either via enemas or orally through a pH-dependent slow release mechanism. However, the results have been inconsistent thus far, and further research with a larger control group is required (Gill et al., 2018). It has been clearly established that short chain fatty acids are produced mainly in the colon as a result of microbial metabolism of non-digestible carbohydrates (Alexander et al., 2019). *Lactobacillus* and *Bifidobacterium*, the main genera used as probiotics, have been shown to produce acetate *in vitro*, however, they were not able to produce propionate and butyrate as primary metabolites. However, the administration of these common probiotics may stimulate the short chain fatty acid production of other colon bacteria through generating pyruvate and lactate from the dietary carbohydrates (LeBlanc et al., 2017). Moreover, probiotics, prebiotics and/or synbiotics can modulate the gut microbiota, and thereby inhibit pathogens and promote the growth of SCFA producing bacteria such as the propionate-producing genera *Bacteriodes, Akkermansia* and butyrate-producing genera such as *Clostridium*, *Lachnospira*, *Roseburia* and several others depending on the (probiotic) strain administered and the targeted disease (Louis and Flint, 2017).

It was shown previously that abundance of the genus *Akkermansia* is significantly decreased in the gut after the consumption of alcohol in an alcoholic hepatitis murine model (Lowe et al., 2017). Similar effects were confirmed in our experiments. The decrease in *Akkermansia* was significantly reversed by the administration of GTB1^TM^ compared to all the other control groups. Strains belonging to the genus *Akkermansia*, a member of the phylum *Verrucomicrobia*, are strictly anaerobic, oval-shaped, Gram-negative, mucin-degrading bacteria, and are highly abundant in the colon of a healthy person, comprising about 1–4% of the total population (Belzer and de Vos, 2012). *Akkermansia* is known to produce acetate (Derrien et al., 2004) and propionate through the fermentation of indigestible carbohydrates by the succinate pathway (Reichardt et al., 2014; Louis and Flint, 2017). The increase of *Akkermansia* therefore may explain the increase of propionate in the AP group (animals receiving GTB1^TM^); this was indicated by the significant decrease in the acetate/propionate ratio (Figure 3E). There have been very few publications that suggest some corelation of *Akkermansia* with proinflammatory cytokines (Collado et al., 2012) and colorectal cancer (Weir et al., 2013). However, potential pathogenicity of *Akkermansia* has not been clearly defined. Furthermore, more studies prove that the decrease of *Akkermansia* was clearly correlated with diabetes, obesity and inflammatory bowel disease demonstrating a protective role of *Akkermansia* and also suggesting it as a promising probiotic (Zhang et al., 2019). The short chain fatty acids, acetate, propionate and butyrate are produced by bacterial fermentation of indigestible carbohydrates within the gut (Morrison and Preston, 2016). Liu et al. (2017) reported that although the administration of propionate did not show attenuation of gastric erosion in the alcohol induced model, it is widely known for its immunomodulatory effects in various gastrointestinal diseases, e.g. by inhibiting pro-inflammatory cytokines such as TNF-α, IL8 and IL6 in inflammatory bowel disease (Tedelind et al., 2007). Apart from the gut, the production of propionate influences the host metabolic health by decreasing lipid synthesis within the liver, regulating satiety through the stimulation of leptin and glucagon-like peptide-1, and lowering serum cholesterol levels (Hosseini et al., 2011; Park et al., 2018).

The GTB1^TM^ treated group showed significant differences in beta-diversity between the microbiota groups in both un-weighted and weighted PCoA plots (Figures 4D,E) demonstrating a different abundance and variety of the microbial communities between each group. Compared to the EtOH control group there was also a significantly higher richness shown through chao1 and the observed OTUs (observed operational taxonomic units) (Figures 4A,B) in the AP group, but a much lower evenness calculated by Shannon diversity. Also, in comparison with the EtOH control group, the richness in the non-treated PBS control group and the OMPZ group (Omeprazole treated) significantly increased (Figure 4C). Loss of microbial diversity is used as one of the indicators of intestinal dysbiosis, especially in metabolic diseases caused by “Western Diet” which are typically characterized by a high amount of fat and sugar (Mosca et al., 2016). The AP group (receiving GTB1^TM^) showed an increase in richness. This suggests significant changes in particular microbial communities, and thereby an overall increase in alpha diversity. A similar change was detected in the OMPZ group. On the other hand, there was only a decrease in evenness of the GTB1^TM^ group, while for all the other groups significant changes could not be detected. Although there was a higher richness in the microbial genera of the AP group, the significantly lower evenness may have been mainly due to the increased ratio of *Akkermansia* within the community (Figure 5B).

Furthermore, the beneficial effects of GTB1^TM^ administration were supported by histological analysis under the light microscope showing a morphologically significant lower gastric tissue erosion level an a significantly reduced pathological score. This result was in fact comparable to that of the OMPZ group, receiving omeprazole, the most commonly used drug for gastric ulcers (Figure 1). In both the GTB1^TM^ and OMPZ treated groups the production of the anti-inflammatory cytokine IL10 (Figure 2A), was increased, while only in the GTB1^TM^ group a lower production of the pro-inflammatory cytokine IL6 (Figure 2B) was detected. The IL6/IL10 ratio (Figure 2C) in the GTB1^TM^ group was significantly reduced compared to the EtOH group. An increase in the ratio of IL6/IL10 has been associated with gastric cancer prognosis (Madej-Michniewicz et al., 2015). Moreover, an increase in IL-6 in association with *H. pylori* pathogenicity and other peptic and duodenal ulcers has also been reported (Sugimoto et al., 2010; Cadamuro et al., 2014; Michalkiewicz et al., 2015).

In this study, we observed the preventive effects in ameliorating alcohol induced gastric erosion through the pretreatment with GTB1^TM^. This pretreatment modulated the gut microbiota by increasing the diversity of the microbial community and the abundance of *Akkermansia* within the gut which in turn increased the ratio of acetate/propionate. The administration of GTB1^TM^ also increased the production of the anti-inflammatory cytokine IL10 concomitantly with a decrease in IL6 production and an overall lowering in the inflammation of the gastric tissue. Our study showed that *Lb. plantarum* strain GTB1^TM^ displays potential probiotic functions through the modulation of the gut microbiota and amelioration of gastric erosion in an alcohol induced murine model.

## Data Availability Statement

The datasets generated for this study are available on request to the corresponding author.

## Ethics Statement

The animal study was conducted at Handong Global University and was approved by the Animal Ethics Committee of Handong Global University, South Korea (20160616-007).

## Author Contributions

HP initiated and performed the experiments supported by EH. YJ and WH guided the initiation and planning of the work and the initial writing of the text together with HP. YJ, ST, and WH collaborated with HP in the interpretation of the data, and in the writing and final editing of the manuscript. YJ, WH, HP, and DC, participated in the experimental design. DC, JS, and WK worked on receiving the funding for this project.

## Conflict of Interest

DC, JS, and WK were employed by the company Vital Beautie, South Korea. The remaining authors declare that the research was conducted in the absence of any commercial or financial relationships that could be construed as a potential conflict of interest.

## References

[B1] Alexander C. Swanson K. S. Fahey G. C. Jr. Garleb K. A. (2019). Perspective: physiologic importance of short-chain fatty acids from nondigestible carbohydrate fermentation. *Adv. Nutr.* 10 576–589. 10.1093/advances/nmz004 31305907 PMC6628845

[B2] Arellano K. Vazquez J. Park H. Lim J. Ji Y. Kang H. J. (2019). Safety evaluation and whole genome annotation of *Lactobacillus plantarum* strains from different sources with special focus on isolates from green tea. *Probiotics Antmicrob. Proteins* [Epub ahead of print]. 10.1007/s12602-019-09620-y 31786735

[B3] Azhari H. Underwood F. King J. Coward S. Shah S. Ng S. (2018). A36, the global incidence of peptic ulcer disease and its complications at the turn of the 21st century: a systematic review. *J. Can. Assoc. Gastroenterol.* 113 684–685. 10.1093/jcag.gwy009.036

[B4] Belzer C. de Vos W. M. (2012). Microbes inside – from diversity to function: the case of *Akkermansia*. *ISME J.* 6 1449–1458. 10.1038/ismej.2012.6 22437156 PMC3401025

[B5] Bergström A. Licht T. R. Wilcks A. Andersen J. B. Schmidt L. R. Grønlund H. A. (2012). Introducing GUt Low-Density Array (GULDA)–a validated approach for qPCR-based intestinal microbial community analysis. *FEMS Microbiol. Lett.* 337 38–47. 10.1111/1574-6968.12004 22967145

[B6] Boltin D. (2016). Probiotics in *Helicobacter pylori*-induced peptic ulcer disease. *Best Pract. Res. Clin. Gastroenterol.* 30 99–109. 10.1016/j.bpg.2015.12.003 27048901

[B7] Bull-Otterson L. Feng W. Kirpich I. Wang Y. Qin X. Liu Y. (2013). Metagenomic analyses of alcohol induced pathogenic alterations in the intestinal microbiome and the effect of *Lactobacillus rhamnosus* GG treatment. *PLoS One* 8:e53028. 10.1371/journal.pone.0053028 23326376 PMC3541399

[B8] Cadamuro A. C. T. Rossi A. F. T. Maniezzo N. M. Silva A. E. (2014). *Helicobacter pylori* infection: host immune response, implications on gene expression and microRNAs. *World J. Gastroenterol.* 20 1424–1437. 10.3748/wjg.v20.i6.1424 24587619 PMC3925852

[B9] Chen Y. Yang F. Lu H. Wang B. Chen Y. Lei D. (2011). Characterization of fecal microbial communities in patients with liver cirrhosis. *Hepatology* 54 562–572. 10.1002/hep.24423 21574172

[B10] Collado M. C. Laitinen K. Salminen S. Isolauri E. (2012). Maternal weight and excessive weight gain during pregnancy modify the immunomodulatory potential of breast milk. *Pediatr. Res.* 72 77–85. 10.1038/pr.2012.42 22453296

[B11] Delzenne N. M. Neyrinck A. M. Bäckhed F. Cani P. D. (2011). Targeting gut microbiota in obesity: effects of prebiotics and probiotics. *Nat. Rev. Endocrinol.* 7 639–646. 10.1038/nrendo.2011.126 21826100

[B12] Derrien M. Vaughan E. E. Plugge C. M. de Vos W. M. (2004). *Akkermansia muciniphila* gen. nov., sp. nov., a human intestinal mucin-degrading bacterium. *Int. J. Syst. Evolut. Microbiol.* 54 1469–1476. 10.1099/ijs.0.02873-0 15388697

[B13] Dharmani P. De Simone C. Chadee K. (2013). The probiotic mixture VSL# 3 accelerates gastric ulcer healing by stimulating vascular endothelial growth factor. *PLoS One* 8:e58671. 10.1371/journal.pone.0058671 23484048 PMC3590171

[B14] Ducrotté P. Sawant P. Jayanthi V. (2012). Clinical trial: *Lactobacillus plantarum* 299v (DSM 9843) improves symptoms of irritable bowel syndrome. *World J. Gastroenterol.* 18 4012–4018. 10.3748/wjg.v18.i30.4012 22912552 PMC3419998

[B15] Engen P. A. Green S. J. Voigt R. M. Forsyth C. B. Keshavarzian A. (2015). The gastrointestinal microbiome: alcohol effects on the composition of intestinal microbiota. *Alcohol Res. Curr. Rev.* 37 223–236. 10.35946/arcr.v37.2.07 PMC4590619 26695747

[B16] Erben U. Loddenkemper C. Doerfel K. Spieckermann S. Haller D. Heimesaat M. M. (2014). A guide to histomorphological evaluation of intestinal inflammation in mouse models. *Int. J. Clin. Exper. Pathol.* 7 4557–4576. 25197329 PMC4152019

[B17] Food and Agriculture Organization [FAO], World Health Organization [WHO] (2002). *Health and Nutritional Properties of Probiotics in Food Including Powder Milk with Live Lactic Acid Bacteria. Report of a joint FAO/WHO Expert Consultation on Evaluation of Health and Nutritional Properties of Probiotics in Food Including Powder Milk With Live Lactic Acid Bacteria.* Available at: http://www.fao.org/3/a-a0512e.pdf. (accessed August 7, 2019).

[B18] Gerritsen J. Smidt H. Rijkers G. T. de Vos W. M. (2011). Intestinal microbiota in human health and disease: the impact of probiotics. *Gen. Nutr.* 6 209–240. 10.1007/s12263-011-0229-7 21617937 PMC3145058

[B19] Gill P. A. Van Zelm M. C. Muir J. G. Gibson P. R. (2018). Short chain fatty acids as potential therapeutic agents in human gastrointestinal and inflammatory disorders. *Aliment. Pharmacol. Ther.* 48 15–34. 10.1111/apt.14689 29722430

[B20] Goderska K. Pena S. A. Alarcon T. (2018). *Helicobacter pylori* treatment: antibiotics or probiotics. *Appl. Microbiol. Biotechnol.* 102 1–7. 10.1007/s00253-017-8535-7 29075827 PMC5748437

[B21] Gomes A. C. Bueno A. A. de Souza R. G. M. Mota J. F. (2014). Gut microbiota, probiotics and diabetes. *Nutr. J.* 13:60. 10.1186/1475-2891-13-60 24939063 PMC4078018

[B22] Gomi A. Harima-Mizusawa N. Shibahara-Sone H. Kano M. Miyazaki K. Ishikawa F. (2013). Effect of *Bifidobacterium bifidum* BF-1 on gastric protection and mucin production in an acute gastric injury rat model. *J. Dairy Sci.* 96 832–837. 10.3168/jds.2012-5950 23200466

[B23] Gotteland M. Brunser O. Cruchet S. (2006). Systematic review: are probiotics useful in controlling gastric colonization by *Helicobacter pylori*. *Aliment. Pharmacol. Therap.* 23 1077–1086. 10.1111/j.1365-2036.2006.02868.x 16611267

[B24] Hosseini E. Grootaert C. Verstraete W. Van de Wiele T. (2011). Propionate as a health-promoting microbial metabolite in the human gut. *Nutr. Rev.* 69 245–258. 10.111/j.1753-4887.2011.00388.x 21521227

[B25] Imhann F. Bonder M. J. Vila A. V. Fu J. Mujagic Z. Vork L. (2017). Proton pump inhibitors affect the gut microbiome. *Gut Microb.* 65 740–748. 10.1136/gutnjnl-2015-310376 PMC4853569 26657899

[B26] Ji Y. Park S. Park H. Hwang E. Shin H. Pot B. (2018). Modulation of active gut microbiota by *Lactobacillus rhamnosus* GG in a diet induced obesity murine model. *Front. Microbiol.* 9:710 10.3389/fmicb.2018.00710 PMC5902571 29692770

[B27] Khoder G. Al-Menhali A. A. Al-Yassir F. Karam S. M. (2016). Potential role of probiotics in the management of gastric ulcer. *Experim. Therap. Med.* 12 3–17. 10.3892/etm.2016.3293 27347010 PMC4906699

[B28] Konturek P. C. Konturek S. J. Brzozowski T. (2009). *Helicobacter pylori* infection in gastric cancerogenesis. *Acta Physiol. Polonica* 12 3–21. 19826177

[B29] Lam E. K. Tai E. K. Koo M. W. Wong H. P. Wu W. K. Yu L. (2007). Enhancement of gastric mucosal integrity by *Lactobacillus rhamnosus* GG. *Life Sci.* 80 2128–2136. 10.1016/j.lfs.2007.03.01 17499310

[B30] LeBlanc J. G. Chain F. Martín R. Bermúdez-Humarán L. G. Courau S. Langella P. (2017). Beneficial effects on host energy metabolism of short-chain fatty acids and vitamins produced by commensal and probiotic bacteria. *Microb. Cell Factor.* 16:79. 10.1186/s12934-017-0691-z 28482838 PMC5423028

[B31] Lee J. U. Kim S. H. Park T. W. Kim O. (2006). Establishment of ethanol-pretreating animal model to study *Helicobacter pylori* infection. *Korea. J. Vet. Res.* 46 327–335.

[B32] Liu J. Wang F. Luo H. Liu A. Li K. Li C. (2016). Protective effect of butyrate against ethanol-induced gastric ulcers in mice by promoting the anti-inflammatory, anti-oxidant and mucosal defense mechanisms. *Int. Immunopharmacol.* 30 179–187. 10.1016/j.intimp.2015.11.018 26604089

[B33] Liu J. Wang J. Shi Y. Su W. Chen J. Zhang Z. (2017). Short chain fatty acid acetate protects against ethanol-induced acute gastric mucosal lesion in mice. *Biol. Pharm. Bull.* 40 1439–1446. 10.1248/bpb.b17-00240 28867726

[B34] Louis P. Flint H. J. (2017). Formation of propionate and butyrate by the human colonic microbiota. *Environ. Microbiol.* 19 29–41. 10.1111/1462-2920.13589 27928878

[B35] Lowe P. P. Gyongyosi B. Satishchandran A. Iracheta-Vellve A. Ambade A. Kodys K. (2017). Alcohol-related changes in the intestinal microbiome influence neutrophil infiltration, inflammation and steatosis in early alcoholic hepatitis in mice. *PLoS One* 12:e0174544. 10.1371/journal.pone.0174544 28350851 PMC5370121

[B36] Madej-Michniewicz A. Budkowska M. Sałata D. Dołęgowska B. Starzyńska T. Błogowski W. (2015). Evaluation of selected interleukins in patients with different gastric neoplasms: a preliminary report. *Sci. Rep.* 5:14382. 10.1038/srep14382 26486258 PMC4613562

[B37] Michalkiewicz J. Helmin-Basa A. Grzywa R. Czerwionka-Szaflarska M. Szaflarska-Poplawska A. Mierzwa G. (2015). Innate immunity components and cytokines in gastric mucosa in children with *Helicobacter pylori* infection. *Mediators Inflamm.* 2015:176726. 10.1155/2015/176726 25948881 PMC4407632

[B38] Morrison D. J Preston T. (2016). Formation of short chain fatty acids by the gut microbiota and their impact on human metabolism. *Gut Microbes* 7 189–200. 10.1080/19490976.2015.1134082 26963409 PMC4939913

[B39] Mosca A. Leclerc M. Hugot J. P. (2016). Gut microbiota diversity and human diseases: should we reintroduce key predators in our ecosystem? *Front. Microbiol.* 7:455. 10.3389/fmicb.2016.00455 27065999 PMC4815357

[B40] Mutlu E. A. Gillevet P. M. Rangwala H. Sikaroodi M. Naqvi A. Engen P. A. (2012). Colonic microbiome is altered in alcoholism. *Am. J. Physiol. Gastroint. Liver Physiol.* 302 966–978. 10.1152/ajpgi.00380.2011 22241860 PMC3362077

[B41] Park S. Ji Y. Jung H. Y. Park H. Kang J. Choi S. H. (2017). *Lactobacillus plantarum* HAC01 regulates gut microbiota and adipose tissue accumulation in a diet-induced obesity murine model. *Appl. Microbiol. Biotechnol.* 101 1605–1614. 10.1007/s00253-016-7953-2 27858139

[B42] Park S. Kang J. Choi S. Park H. Hwang E. Kang Y. (2018). Cholesterol-lowering effect of *Lactobacillus rhamnosus* BFE5264 and its influence on the gut microbiome and propionate level in a murine model. *PLoS One* 13:e0203150 10.1371/jornal.pone.023150 PMC6112659 30153290

[B43] Reichardt N. Duncan S. H. Young P. Belenguer A. McWilliam Leitch C. Scott K. P. (2014). Phylogeneticdistribution of three pathways for propionate productionwithin the human gut microbiota. *ISME J.* 8 1323–1335. 10.1038/ismej.2014.14 24553467 PMC4030238

[B44] Schwiertz A Taras D Schäfer K Beijer S Bos N. A Donus C (2010). Microbiota and SCFA in lean and overweight healthy subjects. *Obesity* 18 190–195. 10.1038/oby.2009.167 19498350

[B45] Şenol K. Özkan M. B. Vural S. Tez M. (2014). The role of inflammation in gastric cancer. *Inflamm. Cancer.* 2014 235–257. 10.1007/978-3-0348 24818726

[B46] Singh P. K. Kaur I. P. (2012). Synbiotic (probiotic and ginger extract) loaded floating beads: a novel therapeutic option in an experimental paradigm of gastric ulcer. *J. Phar. Pharmacol.* 64 207–217. 10.111/j.2042.7158.2011.01397.x 22221096

[B47] Sugimoto M. Yamaoka Y. Furuta T. (2010). Influence of interleukin polymorphisms on development of gastric cancer and peptic ulcer. *World J. Gastroenterol.* 16 1188–1200. 10.3748/wjg.v16.i10.1188 20222161 PMC2839170

[B48] Tedelind S. Westberg F. Kjerrulf M. Vidal A. (2007). Anti-inflammatory properties of the short-chain fatty acids acetate and propionate: a study with relevance to inflammatory bowel disease. *World J. Gastroenterol.* 13 2826–2832. 10.3748/wjg.v13.i20,2826 17569118 PMC4395634

[B49] Toljamo K. Niemelä S. Karvonen A. L. Karttunen R. Karttunen T. J. (2011). Histopathology of gastric erosions association with etiological factors and chronicity. *Helicobacter* 16 444–451. 10.111/j.1523-5378.2011.00871.x 22059395

[B50] Uchida M. Kurakazu K. (2004). Yogurt containing *Lactobacillus gasseri* OLL2716 exerts gastroprotective action against acute gastric lesion and antral ulcer in rats. *J. Pharmacol. Sci.* 96 84–90. 10.1254/jphs.fpj04027X 15359087

[B51] Uchida M. Shimizu K. Kurakazu K. (2010). Yogurt containing *Lactobacillus gasseri* OLL 2716 (LG21 yogurt) accelerated the healing of acetic acid-induced gastric ulcer in rats. *Biosci. Biotechnol Biochem.* 74 1891–1894. 10.1271/bbb.100287 20834166

[B52] Virchenko O. V. Falalyeyeva T. M. Beregova T. V. Maryana S. Y. (2015). The multistrain probiotic enhances the healing process of stress-induced lesions of the gastric mucosa of rats. *Res. J. Pharm. Biol. Chem. Sci.* 6 249–259.

[B53] Wang F. Y. Liu J. M. Luo H. H. Liu A. H. Jiang Y. (2015). Potential protective effects of *Clostridium butyricum* on experimental gastric ulcers in mice. *World J. Gastroenterol.* 21 8340–8351. 10.3748/wjg.v21.i27.8340 26217085 PMC4507103

[B54] Weir T. L. Manter D. K. Sheflin A. M. Barnett B. A. Heuberger A. L. Ryan E. P. (2013). Stool microbiome and metabolome differences between colorectal cancer patients and healthy adults. *PLoS One* 8:e70803. 10.1371/journal.pone.0070803 23940645 PMC3735522

[B55] Zhang T. Li Q. Cheng L. Buch H. Zhang F. (2019). Akkermansia muciniphila is a promising probiotic. *Microb. Biotechnol.* 12 1109–1125. 10.1111/1751-7915.13410 31006995 PMC6801136

