# Supplementary material for: Amelioration of Alcohol Induced Gastric Ulcers Through the Administration of Lactobacillus plantarum APSulloc 331261 Isolated From Green Tea
Source: Front Microbiol. 2020 Mar 17;11:420. doi: 10.3389/fmicb.2020.00420 (PMC7090068; doi:10.3389/fmicb.2020.00420)
Supplement: Supplementary file 1 [file Image_1.pdf]

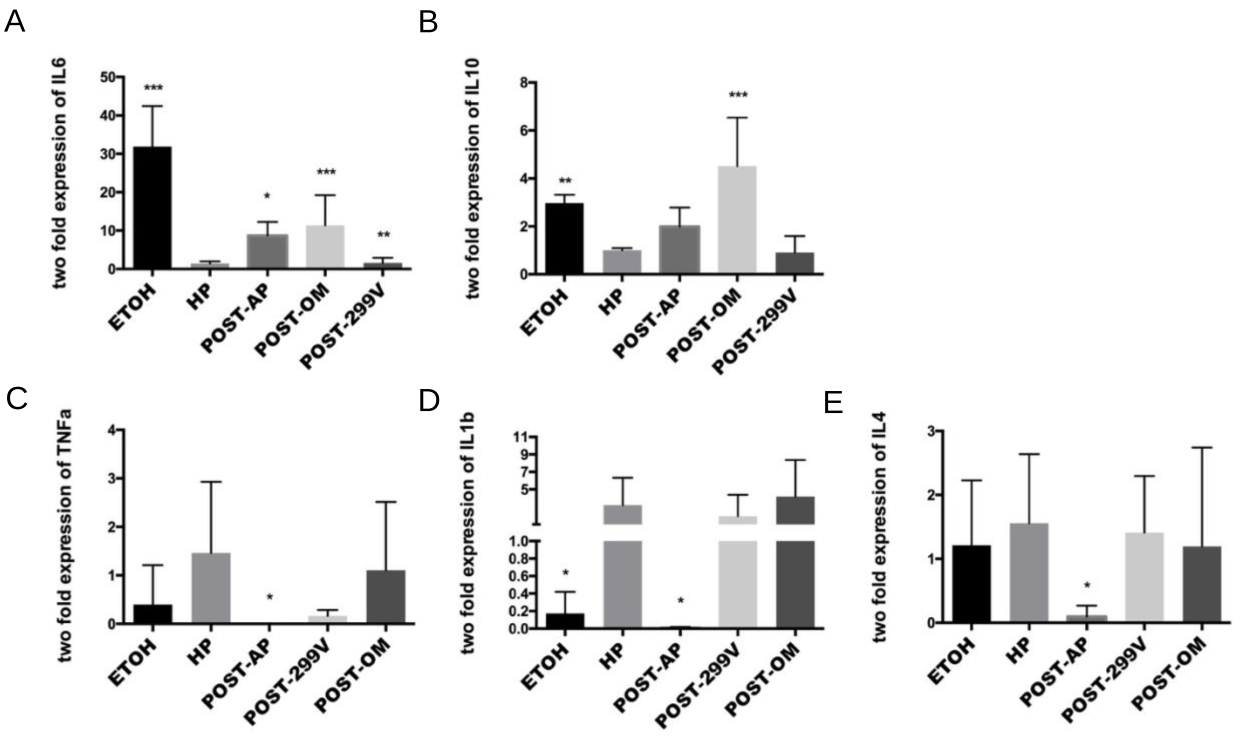

FIGURE S1 The effect of *Lactobacillus plantarum* APSulloc 331261 GTB1™ on IL10, IL6, TNFa, IL1b, IL4 levels in gastric tissue from the *H. pylori* infected therapeutic mouse model (**A**) IL6: interleukin 6 (**B**) IL10: interleukin 10 (**C**) TNFa: tumor necrosis factor alpha (**D**) IL1b: interleukin 1 beta (**E**) IL4: interleukin 4. ETOH: ethanol treated control group, HP: *H. pylori* infected group, OM: omeprazole treated group, 299v: *Lactobacillus plantarum* 299v, AP: *Lactobacillus plantarum* APSulloc 331261 GTB1™. Data was analyzed with one-way-ANOVA compared to EtOH; *p<0.05, **p<0.01, ***p<0.001.
