# Supplementary material for: Amelioration of Alcohol Induced Gastric Ulcers Through the Administration of Lactobacillus plantarum APSulloc 331261 Isolated From Green Tea
Source: Front Microbiol. 2020 Mar 17;11:420. doi: 10.3389/fmicb.2020.00420 (PMC7090068; doi:10.3389/fmicb.2020.00420)
Supplement: Supplementary file 2 [file Image_2.pdf]

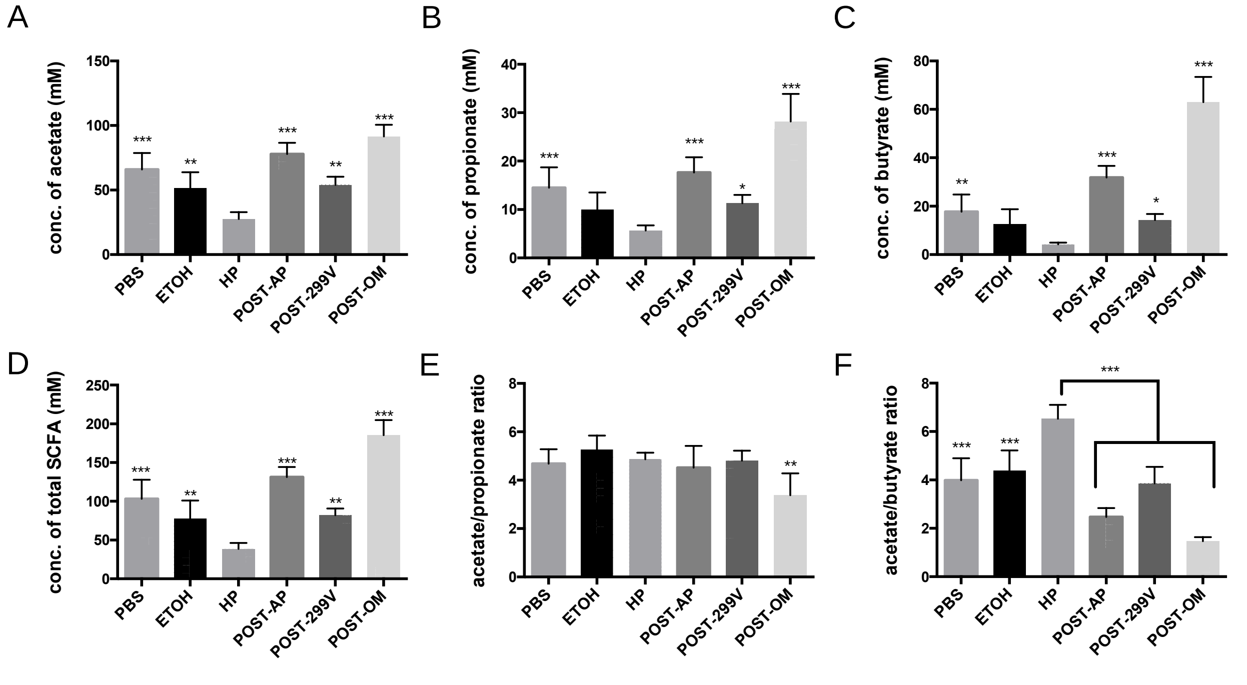

FIGURE S2 The production of SCFAs in the cecum through the therapeutic treatment of *Lactobacillus plantarum* APSulloc 331261 GTB1^{TM} in *H. pylori* infected mice. (**A**) Acetate (**B**) Propionate (**C**) Butyrate (**D**) Total SCFAs (**E**) Acetate/propionate ratio (**F**) Acetate/butyrate ratio*.* ETOH: ethanol treated control group, HP: *H. pylori* infected group, OM: omeprazole treated group, 299v: *Lactobacillus plantarum* 299v, AP: *Lactobacillus plantarum* APSulloc 331261 GTB1^{TM}. Data was analyzed with one-way-ANOVA compared to EtOH *$p<0.05$, **$p<0.01$, ***$p<0.001$.
