# Supplementary material for: Amelioration of Alcohol Induced Gastric Ulcers Through the Administration of Lactobacillus plantarum APSulloc 331261 Isolated From Green Tea
Source: Front Microbiol. 2020 Mar 17;11:420. doi: 10.3389/fmicb.2020.00420 (PMC7090068; doi:10.3389/fmicb.2020.00420)
Supplement: Supplementary file 3 [file Image_3.pdf]

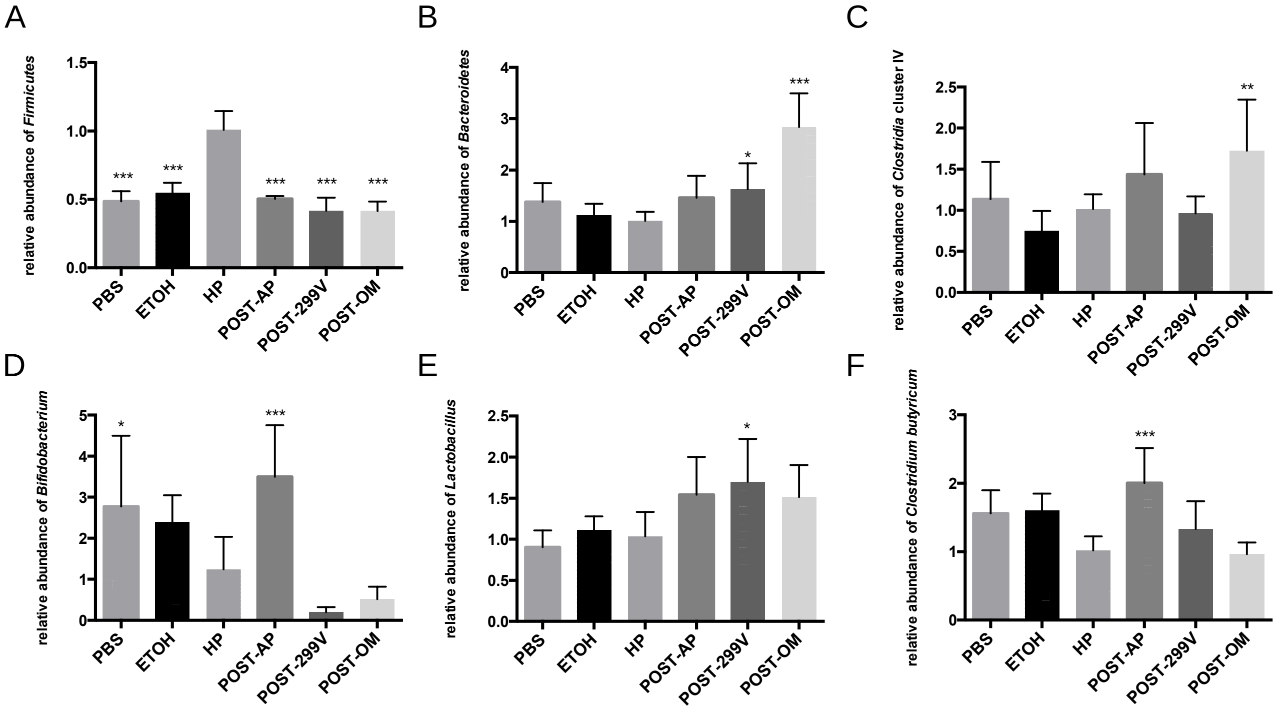

FIGURE S3 Modulation of the gut microbiota in the cecum through the therapeutic treatment of *Lactobacillus plantarum* APSulloc 331261 GTB1TM in *H. pylori* infected mice. (**A**) *Firmicutes,* (**B**) *Bacteroidetes,* (**C**) *Clostridium* cluster IV, (**D**) *Bifidobacterium* species, (**E**) *Lactobacillus* species, (**F**) *Clostridium butyricum.* ETOH: ethanol treated control group, HP: *H. pylori* infected group, OM: omeprazole treated group, 299v: *Lactobacillus plantarum* 299v, AP: *Lactobacillus plantarum* APSulloc 331261 GTB1TM. Data was analyzed with one-way- ANOVA compared to EtOH. *p<0.05, **p<0.01, ***p<0.001.
