# Supplementary material for: Amelioration of Alcohol Induced Gastric Ulcers Through the Administration of Lactobacillus plantarum APSulloc 331261 Isolated From Green Tea
Source: Front Microbiol. 2020 Mar 17;11:420. doi: 10.3389/fmicb.2020.00420 (PMC7090068; doi:10.3389/fmicb.2020.00420)
Supplement: Supplementary file 4 [file Table_1.DOCX]

**Supplementary Data**

**Table S1. Primer list for the qRT-PCR analysis of the gastric tissue**

| **Primer** | **Forward** | **Reverse** |
| --- | --- | --- |
| GAPDH | TGTGTCCGTCGTGGATCTGA | CCTGCTTCACCACCTTCTTGA |
| IL6 | CTGCAAGAGACTTCCATCCAGTT | AAGTAGGGAAGGCCGTGGTT |
| IL10 | TGCTATGCTGCCTGCTCTTAC | CGGTTAGCAGTATGTTGTCCAG |
| TNFα | ACTGCCAGAAGAGGCACTCC | CGATCACCCCGAAGTTCA |
| IL1b | GACCTTCCAGGATGAGGACA | AGCTCATATGGGTCCGACAG |
